# Investigating the effects of brain stimulation on the neural substrates of inhibition in patients with OCD: A simultaneous tDCS – fMRI study

**DOI:** 10.1038/s41398-025-03381-9

**Published:** 2025-05-19

**Authors:** Daniela Rodriguez-Manrique, Hanyang Ruan, Chelsea Winkelmann, Julian Haun, Sandra Gigl, Götz Berberich, Claus Zimmer, Kathrin Koch

**Affiliations:** 1 Department of Diagnostic and Interventional Neuroradiology, School of Medicine, Technical University of Munich, Munich, Germany; 2 Neuroimaging Centre (TUM-NIC), Klinikum rechts der Isar, Technical University of Munich, Munich, Germany; 3 Graduate School of Systemic Neurosciences, Ludwig Maximilians University Munich, Martinsried, Germany; 4 Windach Institute and Hospital of Neurobehavioural Research and Therapy (WINTR), Windach, Germany

**Keywords:** Psychiatric disorders, Human behaviour, Predictive markers

## Abstract

Inhibition deficits constitute a core characteristic of obsessive-compulsive disorder (OCD). There is evidence in healthy individuals that transcranial direct current stimulation (tDCS) of the pre-supplementary motor area (pre-SMA) leads to a significantly improved inhibition performance. Against this background we investigated the effects of pre-SMA tDCS on inhibition performance and the underlying neural correlates in patients with OCD. Using a double-blind, randomized, sham-controlled, cross-over design (i.e., tDCS sham vs. tDCS stimulation) we investigated the effects of 2 mA anodal tDCS stimulation of the right pre-SMA in a sample of 47 OCD patients. The present study is, to our best knowledge, the first study applying concurrent tDCS-fMRI in patients with OCD. tDCS was applied using the MRI-compatible NeuroConn DC-Stimulator which allowed for a concurrent stimulation, while patients performed an inhibition (i.e., Stroop) task in a 3 T MRI. Imaging data were analysed using a multivariate partial least squares (PLS) approach. tDCS stimulation (vs. sham) was associated with increased activation in a fronto-parieto-cerebellar network comprising, amongst others, the precentral, middle frontal and inferior frontal gyrus, the anterior cingulate and the superior parietal lobe. On the performance level, tDCS stimulation (vs. sham) was linked to an improved inhibition performance in terms of an increased percentage of correct responses in the Stroop task. Present results indicate that tDCS in patients with OCD goes along with an improved inhibition performance as well as activation increases in regions known to be involved in inhibition, motor, and cognitive control. Thus, our findings suggest that tDCS might be a promising method to improve specific impairments in OCD.

## Introduction

Obsessive-compulsive disorder (OCD) is a debilitating psychiatric disorder affecting 1% of the general population [1]. It is characterized by time-consuming obsessions (i.e., repetitive intrusive thoughts) and compulsions (i.e., repetitive behaviours that serve to counteract anxiety caused by obsessive thoughts). One major characteristic of OCD is an impaired response inhibition as measured by, for instance, the Stroop or the stop-signal task (SST) [2–4]. In successful response inhibition one manages to inhibit a specific automatic process (such as, in case of the Stroop task, reading the name of a colour word shown in a colour different from the word or, in case of the SST, performing a motor action in response to the appearance of a stop signal). Problems with inhibition are self-evident in the phenomenology of OCD since patients are obviously unable to properly inhibit their obsessions and compulsions. Accordingly, OCD patients have repeatedly been shown to be impaired in their inhibition performance by demonstrating an increased response time and/or percentage of errors during the Stroop incongruent condition (i.e., when word reading has to be inhibited) [5–7] or an elevated stop-signal reaction time in the SST [4, 8].

In the healthy brain, response inhibition has been shown to go along with increased activation in a network of regions comprising mainly the presupplementary motor area (pre-SMA), the motor cortex including the precentral gyrus, the dorsolateral prefrontal cortex (DLPFC), the medial prefrontal cortex (mPFC), the subthalamic nucleus, the posterior parietal cortex (PPC) and the right inferior frontal gyrus (IFG) [9–11]. Previous studies also reported high activation of IFG [12] and pre‐SMA [13] to be related to good performance on the SST in healthy subjects. In addition, a recent activation likelihood estimation meta-analysis investigating brain activation in association with inhibition performance in the Stroop task [14] pointed at the involvement of a network containing the right cingulate cortex, the left dorsolateral prefrontal cortex, bilateral inferior frontal gyri, the right superior frontal gyrus and the temporal cortex.

Interestingly, the regions found to be altered in OCD patients in association with inhibition performance are only partly overlapping with those regions found to be relevant for inhibition in healthy subjects. Thus, Norman et al. [15] who performed a large meta-analysis on the neural substrates of error processing and inhibitory control comprising data from 239 OCD patients and 229 healthy control subjects found inhibition-related hypoactivation in OCD in the rostral and ventral anterior cingulate cortices, the thalamus/caudate, the anterior insula/frontal operculum, the supramarginal gyrus, and the medial orbitofrontal cortex in association with longer inhibitory control reaction times. Single studies, however, provided also some evidence for a decreased inhibition-related activation in, amongst others, the pre-SMA, the thalamus, the orbitofrontal cortex, the IFG and the striatum in OCD [16–19], but also for an increased activation of pre‐SMA in association with reduced activation of the IFG [20].

Studies in healthy controls indicate that transcranial direct current stimulation (tDCS), a non-invasive brain stimulation treatment that uses direct electrical currents to stimulate specific parts of the brain, might be an effective technique to improve specific cognitive processes including inhibition performance [21, 22]. In healthy controls, tDCS of the pre-SMA has been found to increase inhibition performance in terms of an improvement in inhibiting responses when a stop signal was presented in the SST task [23] as well as in terms of an increased stopping speed along with an increased blood-oxygen level dependent (BOLD) response in the pre-SMA and ventromedial prefrontal cortex (vmPFC) [21].

Surprisingly, comparable studies in OCD have – to our best knowledge - not been performed, up to now. Against this background, in the current study we employed an MRI-compatible tDCS device in a sample of patients suffering from OCD to stimulate the brain while patients were performing the Stroop inhibition task in the MRI. Given the relevance of the pre-SMA for inhibition performance we chose the right pre-SMA as the main target of anodal stimulation. In addition, given previous studies showing that intensity of the stimulation effects (i.e., electric field strength) and individual brain anatomy are critical determinants of the final stimulation effects [24–28], we investigated potential effects of these parameters in the framework of a mediation analysis (for more details please refer to the methods section).

Imaging data were analysed using an event-related approach with partial least squares (PLS) [29], a multivariate analysis technique that identifies whole-brain patterns of covariance related to the experimental design. PLS uses singular value decomposition to classify the fMRI data into orthogonal latent variables (LVs) explaining the maximum amount of covariance between the task conditions and the fMRI signal.

In contrast to the classical mass univariate approach which compares activity independently at each voxel, PLS relies on activity patterns from several voxels. Since PLS is sensitive to magnitude of spatial variability in activation and allows for testing how distributed patterns of BOLD activation across multiple voxels relate to experimental variables, it is often more powerful than the classical univariate approach [30, 31]. Moreover, and even more importantly, PLS is insensitive to interindividual variability in mean brain activation [30]. Given the well-known clinical heterogeneity of OCD and its associated variability in neural activation, there is strong reason to assume the presence of a high interindividual variability in mean brain activation in our patient sample. For the purpose of neutralising the effects of this interindividual variability (which is known to impact detection power also in the context of within-subject designs) we opted for employing the PLS method instead of the classical univariate approach. We expected anodal tDCS over the pre-SMA to go along with both a stimulation-related improvement in inhibition performance as well as an increased activation in regions shown to be relevant for inhibition, such as the pre-SMA and the IFG.

This study set out to study two hypotheses: (1) tDCS as compared to sham stimulation will go along with a significant improvement in response inhibition and (2) tDCS as compared to sham stimulation will go along with an increased activation during inhibition in preSMA and vmPFC. The increased activation will be correlated with an improvement in response inhibition.

## Methods

### Participants

A total of n = 47 patients with OCD as the primary diagnosis according to DSM-5 criteria were included in the study. Of the 54 patients originally recruited, 7 patients were excluded due to study drop-out (n = 1), major artefacts (n = 2), impedance exceedance (n = 2), button-box malfunction (n = 1) task incompletion (n = 1). Adequate power was measured considering studies measuring tDCS effects on Stroop inhibition performance on healthy participants, as there were no studies in OCD patients with comparable design. The necessary sample size was calculated (based on a paired t-test given medium effect sizes of 0.5–0.6). Given a sample size of 39–54 participants, medium size effects (0.5–0.6) can be detected with a reasonable power of 0.95. Based on these considerations, our sample size of 47 patients seems to be adequate. Recruitment took place at several hospitals in and around Munich including the Psychosomatic Clinic in Windach, the Tagesklinik Westend, the Psychiatry at the LMU Clinic and the Schön Klinik Roseneck. All diagnoses were made by an experienced psychiatrist from the respective hospital specialized in the treatment of OCD. Inclusion criteria comprised right-handedness, age 18-65 years, at least a score of 8 in the Y-BOCS scale, willingness, and ability to provide consent and 8 weeks of stable medication/non-medication treatment. Exclusion criteria encompassed neurological disorders (including epilepsy, seizures), psychiatric comorbidities (including schizophrenia, schizo-affective disorder, bipolar disorder, substance abuse and PTSD), incompatibility with MRI scanners (e.g., intracranial implants, pacemakers, or defibrillators), pregnancy, any additional psychopharmacological medication (e.g. antipsychotic medication) and benzodiazepine intake within 24 h of either appointment.

Patients had a mean age of 31.4 (Table 1). n = 14 patients were drug-naive or medication-free for at least 8 weeks. No patients were excluded due to comorbidities and n = 32 patients had one or more comorbid diagnoses. Of those 32 patients, 13 had more than one comorbidity. Comorbidities observed were: depression (n = 19), anxiety (n = 10), eating disorders (n = 3), recurrent depressive disorder (n = 2), light depression (n = 2), Asperger syndrome (n = 2), personality disorder (n = 2), panic disorder (n = 1), dermatillomania (n = 1), social phobia (n = 1), body dysmorphia (n = 1), obsessive-compulsive personality disorder (n = 1), ADHD (n = 1) and adjustment disorder (n = 1). To assess clinical severity of obsessive-compulsive symptoms, patients filled out the self-rated version of the Yale-Brown Obsessive-Compulsive Scale (Y-BOCS) [32] as well as the Y-BOCS checklist. In addition, the Hamilton Depression Rating Scale (HAMD-D) [33] was used to assess depressive and the Hamilton Anxiety Rating Scale (HAM-A) [34] was employed to assess anxiety symptoms. Potential tDCS side-effects were assessed by standardized questionnaires immediately after the tDCS-MRI session (table S1). The present study was approved by the Ethics Committee of the Klinikum rechts der Isar in München and it was in accordance with the Declaration of Helsinki and was registered under https://www.isrctn.com/ISRCTN99571476. All participants gave their informed consent to the study.

**Table 1:** Demographic and clinical data of patients (n = 47).

Category	Description
Sex (male/female)	(32/15)
Age (mean, SD)	(31.4, 11.1)
Duration of illness (mean, SD)	(15.8, 10.3)
Medication (yes/no)	(32/15)
Y-BOCS total (mean, SD)	(20.2, 5.5)
Y-BOCS obsessions (mean, SD)	(10.0, 3.1)
Y-BOCS compulsions (mean, SD)	(10.2, 3.7)
Comorbidity (yes/no)	(33/14)
HAM-D (mean, SD)	(19.9, 9.5)
HAM-A (mean, SD)	(19.8, 9.9)
tDCS side effects	See supplementary table S1

### Image acquisition

Image acquisition was conducted on a 3 T Philips Ingenia (Philips Healthcare, Best, the Netherlands) using a 32-channel (SENSE) head coil. Imaging consisted of a T1-weighted 3D MPRAGE sequence (230 slices, sagittal orientation, 368 ×317 matrix, 0.7 mm isotropic resolution, TR = 11 ms, TE = 5.2 ms, flip angle = 8°), and a T2*-weighted echo-planar imaging (EPI) imaging sequence (TR = 1000 ms, TE = 30 ms, flip angle = 60°, MB factor = 2, matrix size = 64 × 62, field of view = 192 × 192 × 118.5 mm, 64 transverse slices, 3.0 mm slice thickness, whole brain coverage, 3 × 3 × 3 mm^3^ resolution). A series of 660 whole-brain volumes was recorded. In addition, a DTI sequence, another T2* sequence, a resting state fMRI sequence and a FLAIR sequence were acquired in the same imaging session.

### Study design and paradigm

The study was conducted at the Klinikum rechts der Isar, and had a double-blind, randomized, sham-controlled, cross-over design (tDCS sham vs. tDCS stimulation). Randomisation was performed before study initiation, assigning each participant number its group/condition order. Each patient participated in the study on two distinct days, separated by a 7-day interval to avoid any potential carryover effects. Prior to both tDCS-MRI sessions, patients completed a consent form and a comprehensive questionnaire outlining inclusion/exclusion criteria.

All patients underwent a total of around 50–60 min of MRI scanning. The Stroop task was started after the Stop-Signal task, each lasting around 10 min, therefore encompassing the entire tDCS stimulation (or tDCS sham stimulation) duration.

**Fig. 1:**
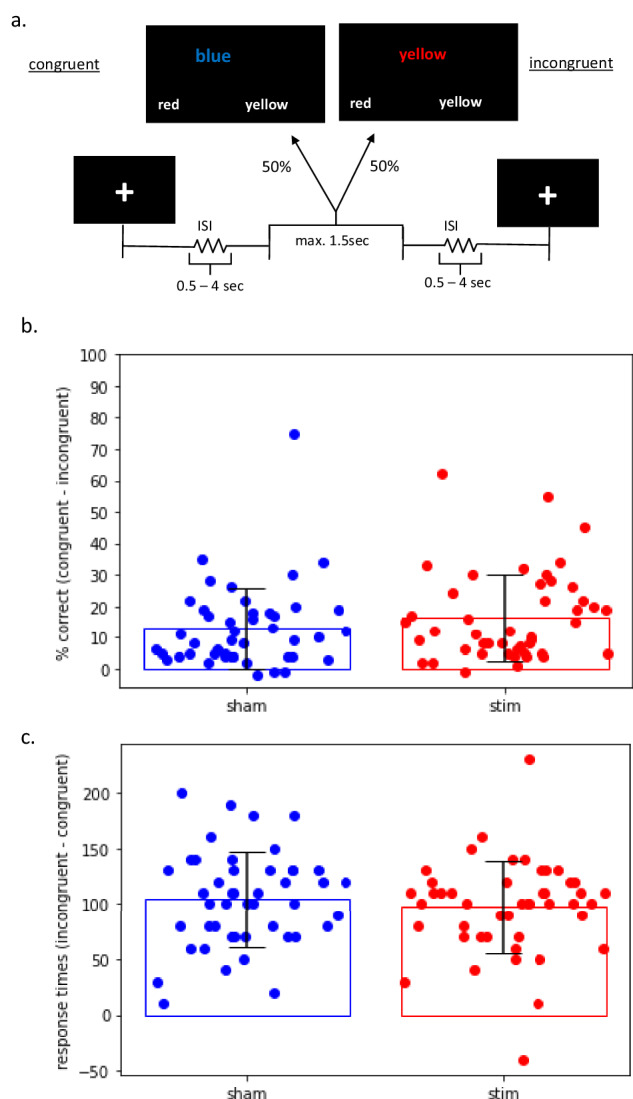
Task Inhibition Measures. **a** Schematic illustration of the Stroop task. **b**, **c** Inhibition performance outlined as % correct (congruent -incongruent) (**b**) and response time in millisecond (incongruent - congruent) (**c**) for sham and stimulation. A non - parametric paired Wilcoxon-signed-rank test was performed to compare performance in the two conditions.

The Stroop task was presented in an event-related design and consisted of a congruent and an incongruent condition. In the congruent condition, colour words were presented in the colour denoted by the corresponding word while in the incongruent condition colour words were displayed in one of three colours not denoted by the word. The target stimulus was presented in the centre of the display screen. Two possible answers were presented below, and patients had to indicate the colour by pressing one of two buttons. Stimuli were presented in 48 congruent and 48 incongruent combinations of four colour words “red,” “green,” “yellow,” and “blue” written in the German language and corresponding colours were presented in a pseudorandom sequence. Stimulus presentation time was 1500 ms with a randomised interstimulus interval of between 0.5 s and 4 s with a mean of 1 s (Fig. 1a). The Stroop task was implemented using Psychopy running on a PC which was connected to a video projector. The stimuli were projected on to a transparent screen inside of the scanner tunnel which could be viewed by the subject through a mirror system mounted on top of the MRI head coil.

### tDCS

The tDCS device that we employed in the current study was the MRI-compatible NeuroConn DC-Stimulator (https://www.neurocaregroup.com/de/technologie/dc-stimulator). To ensure both methodological accuracy (e.g., low impedance) and safety of stimulation application, the experimental set-up inside and outside the scanner was tested extensively, examined by our in-house physicist as well as experts from NeuroConn and adhered strictly to the guidelines provided. For electrode placement optimization targeting the pre-SMA, we employed electrical field calculations using SimNIBS (http://www.simnibs.org). The optimal electrode placement for stimulating the pre-SMA was determined using an EEG 10-20 cap. The anodal stimulation site was located over the centre point of the FC1 with a 3×3 rubber electrode; the cathode was placed over the centre point of the FC2 using a 4×4 rubber electrode. Prior to electrode application, the patient’s hair and scalp were prepared using Ten20 electrode paste to improve skin conductivity underneath the electrodes. The tDCS was configured at a current of 2 mA, with a fade-in and fade-out duration of 15 s, and variable stimulation duration of either 30 s (sham condition) or 1200 s (stimulation condition). Impedance was maintained below 15 Ohm once the 2 mA threshold was reached.

### Data analysis

Performance in the Stroop task was analysed using SPSS 28.0.0.1 (https://www.ibm.com/de-de/products/spss-statistics). To investigate potential performance differences between the two conditions (i.e., Stroop inhibition performance during tDCS stimulation vs. Stroop inhibition performance during tDCS sham) we performed two non-parametric paired Wilcoxon tests for percentage of correct responses (congruent condition – incongruent condition) and mean response times (incongruent condition – congruent condition). Non-parametric tests were employed since data were not normally distributed.

### fMRI preprocessing and analysis

47 patients with OCD were included in the analysis upon excluding participants whose task performance values were outliers. fMRI data were preprocessed using SPM 12. Realignment of images of a functional time series was completed to account for in-scanner head motion. Data was then normalised to a standard template in Montreal Neurological Institute space. This included coregistration, CAT12 segmentation, creation of a DARTEL template based on tissue probability maps and normalisation by DARTEL. Signal to noise ratio was increased by applying a 6 × 6 × 6 kernel gaussian filter. A band-pass filer 0.01–0.08 (or 0.1 Hz) was also applied to the data to remove the frequencies which are not of interest such as noise related to scanner drift, coils, cardiac noise etc. Excessive head motion was established with framewise displacement (FD), calculated as the sum of the absolute values of the derivatives of the 6 motion parameters derived from SPM12 [35].

### Partial least squares analysis

The imaging data recorded during the Stroop task were analysed using a non-rotated event-related partial least squares approach [36]. This multivariate analysis technique identifies whole-brain patterns of covariance related to conditions of an experiment. Each brain voxel has a weight, referred to as salience, which specifies how strongly the voxel contributes to the covariance explained by a so-called latent variable (LV). Each LV contains a pair of vectors relating brain activity and experimental design. In the mean-centred event-related PLS, LVs highlight the dominant patterns of cross-covariance between the fMRI data and task conditions within the mean-centred matrix decomposed with singular value decomposition. For the non-rotated version of the PLS, one examines patterns exclusive to a specific contrast of conditions.

The LVs were determined with a permutation test using 2000 permutations, each event had a temporal window size of 14 time-points (i.e., equivalent to 14 s) post-stimulus onset, called lags. Permutation tests assesses whether the effect represented in each LV can statistically be differentiated from noise. LVs consist of three components: cross-block variance, singular values describing the proportion of covariance of each LV; design-saliences which display the difference between tasks across groups and time and brain saliences, with weights assigned to each voxel at each lag. PLS additionally calculates temporal brain scores which are subject, design-salience, lag and LV specific. These are calculated as the dot product of design-saliences (of a particular LV) and the subjects’ singular brain activity (a data matrix) [37]. Brain scores are similar to factor scores and indicate how strongly individual subjects express the patterns on the LV for each timepoint (lag). To answer our hypothesis on the effect of tDCS on the inhibition (incongruent) condition, we contrasted between task conditions (i.e., incongruent > congruent) and timepoints (tDCS > sham).

Furthermore, the reliability of each voxel’s contribution to a particular LV was tested by submitting all saliences to a bootstrap estimation of the standard errors (SEs), using 2,000 bootstraps. The bootstrap ratio (BSR) is calculated by dividing salience by the SE. Reported are peak voxels with a salience/SE ratio ≥3.0 or ≤−3 for negative correlations (p < 0.001), analogously to z-values ± 3, resulting in a confidence interval of >99% if the bootstrap distribution is normal [37–41]. For the one-sample t-test differentiating between conditions, however, a stricter salience/SE ratio of ≥5.0 or ≤−5 was chosen [40]. This paper reports the peak coordinates from time lags at which the temporal brains score profiles maximally differentiate [36, 42] with a spatial extent threshold of 100 voxels. Of note, lag 6 (i.e., 6 s post stimulus) corresponds to the canonical model of the haemodynamic response function resembling a gamma function peaking at 5 to 6 s following neuronal activation (see supplementary figure S1) [43, 44]. Results based on lags 5, 7 and 8 are reported in the supplementary material (figure S2). The figures were created using ITK-SNAP and ParaView programs [45, 46].

### Electric field modelling

**Fig. 2:**
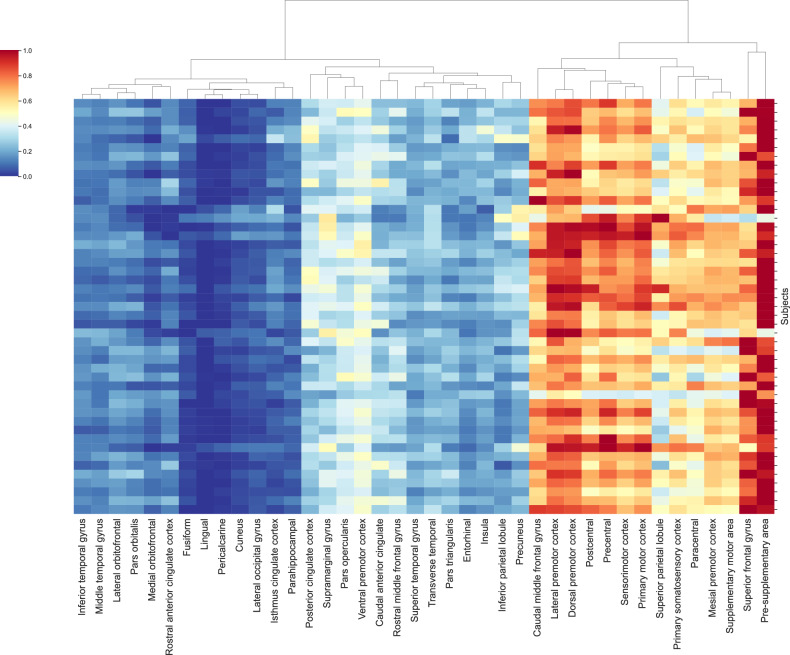
Electric field Magnitude Distribution. Mean electric field at 40 different regions of interest (31 from the Desikan-Killiany-Tourville atlas and 9 from the Human Motor Area Template atlas), averaged across the two sessions, and normalised across all subjects for comparison (n = 47).

The EF on the grey matter was calculated to quantitively assess the variation in dosage between sessions and subjects. The software SimNIBS was used to calculate the EF induced by individual tDCS set-up (charm — SimNIBS documentation). The exact centre coordinates and orientation of the underlying electrode gel were visible on the T1 images and identified using the regionprops MATLAB function. Individual patient T1 and T2-weighted images were segmented and meshed to create tetrahedral head models using SimNIBS: charm [47]. SimNIBS assigns isotropic conductivity values for the fifteen different head tissues assigned in the head models. The two above mentioned electrode rectangular sizes were simulated, with an electrode thickness of 2 mm and a stimulation intensity of −2mA at the anode and 2 mA at the cathode. The EF strength and focality were extracted from the individual grey matter region with field strengths higher than the 99.9th percentile (for an illustration of these parameters see supplementary figure S3). MNI coordinates [−6, 11, 60] with a 10 mm radius were selected as the ROI for the pre-SMA, according to the Human Motor Area Template (HMAT) atlas [48]. This allowed the calculation of the mean EF intensity at the ROI with the SimNIBS python package. The mean EF intensity was measured identically for an additional 31 ROIs taken from the Desikan-Killiany-Tourville (DKT) Atlas and 9 ROIs taken from the HMAT atlas [49] (Fig. 2). Figure 2 was created using python seaborn.clustermap. The cortical surface of each subject was reconstructed using FreeSurfer software with automatic “recon -all” pipeline (version 7.4.0; https://surfer.nmr.mgh.harvard.edu/). This process includes the predefined steps: bias correlation, skull stripping, tissue intensity normalization, Talairach system transformation, and segmentation of grey/white matter. No manual corrections were performed. Subsequently, we extracted the thickness data of the pre-SMA in both hemispheres, as defined by the HMAT atlas [48].

### Mediation analysis

Finally, to investigate the potential influence of the two influencing factors that are known to affect individual tDCS stimulation effects, i.e. EF strength and pre-SMA cortical thickness (for details regarding thickness calculation please refer to the supplement), we performed a mediation analysis to assess potential effects of these influencing factors on stimulation-related changes in inhibition performance. Since there were no significant stimulation-related changes in mean response times (see results section), the mediation analysis was performed only for the percentage of correct responses (% correct). Thus, the stimulation-related change in the percentage of correct responses (i.e., % correct congruent-incongruent for stimulation-sham) constituted the dependent variable, stimulation-related changes in the inhibition-associated brain activation during the Stroop incongruent condition (i.e., PLS brain scores for the incongruent condition for stimulation-sham) the independent variable and EF the mediating variable. Another analogue model was set up with thickness of our anodal stimulation region (i.e., right pre-SMA) as mediating variable. Using these models, we aimed at investigating both a potential indirect (i.e., mediated via EF strength or right pre-SMA thickness) effect of stimulation-related brain activation on stimulation-related inhibition performance as well as a potential direct effect of EF strength or right pre-SMA thickness on stimulation-related inhibition performance. Structural equation modelling was performed using the program Amos 26.0.0 (http://amosdevelopment.com) applying a maximum-likelihood algorithm for estimating path coefficients. We used bootstrapping procedures which make no a priori assumptions about the distribution of the paths. The goodness of fit index (GFI) was used to assess the goodness-of-fit of the two models. Collinearity between brain activation and EF strength (or, respectively, pre-SMA thickness) was checked using linear regression. This yielded no significant results indicating a statistically negligible collinearity between the two variables.

## Results

### Performance data

The paired Wilcoxon test for the percentage of correct responses congruent condition – incongruent condition) yielded a significant result (z = 2.37, n = 47, p = 0.02) indicating a significantly larger percentage of correct responses for the stimulation condition (Fig. 1b). The paired Wilcoxon test for the mean response times (incongruent condition – congruent condition) yielded no significant result (z = 1.27, n = 47, n.s.) (Fig. 1c).

### Imaging data

#### Condition effects

**Fig. 3:**
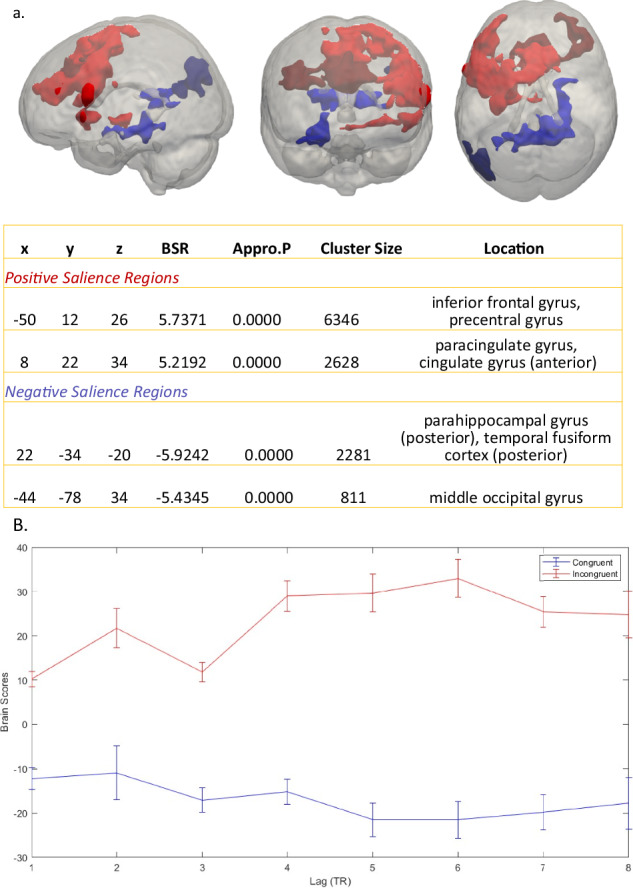
Condition Effects, lag 6. **a** Clusters where peak voxels have a salience/standard error ratio (i.e. Bootstrap ratio; BSR) of ≥5 or ≤−5 for the latent variable (LV) describing 100% of all the variance for our desired contrast comparing incongruent > congruent. Red clusters indicate increased activation for the incongruent condition, blue clusters illustrate increased activation for the congruent condition. Lag 6 represents the brain activity at 6 TRs post stimulus onset (i.e., 6 s post stimulus given our sequence with a TR of 1 s). **B** Temporal brain scores plot displaying the mean brain scores and standard deviation across all 47 subjects for each condition during the first 8 TRs post stimulus onset. Brain scores are subject, design-salience, lag and LV contrast specific. Note the positive and negative peaks are at around 5–6 s after stimulus onset.

A mean-centred non-rotated FPLS was performed to examine, in a first step, the condition effects comparing incongruent vs. congruent trials, independent of timepoint (i.e., stimulation condition). The PLS found a significant condition-LV (p < 0.001), where the incongruent condition of both timepoints had positive brain scores, indicating a greater activity for the incongruent condition (shown in red in the singular image of lag 6, Fig. 3). The congruent conditions had negative brain scores, which were significantly different to the incongruent condition (p < 0.05), thus indicating that for the congruent condition activity was greater in the negative brain salience regions (shown in blue in the singular image of lag 6, Fig. 3). Figure 3 illustrating activation across the different lags shows, for our condition of interest (i.e., incongruent condition), largest activation for lag 6.

#### Condition-by-timepoint effects

**Fig. 4:**
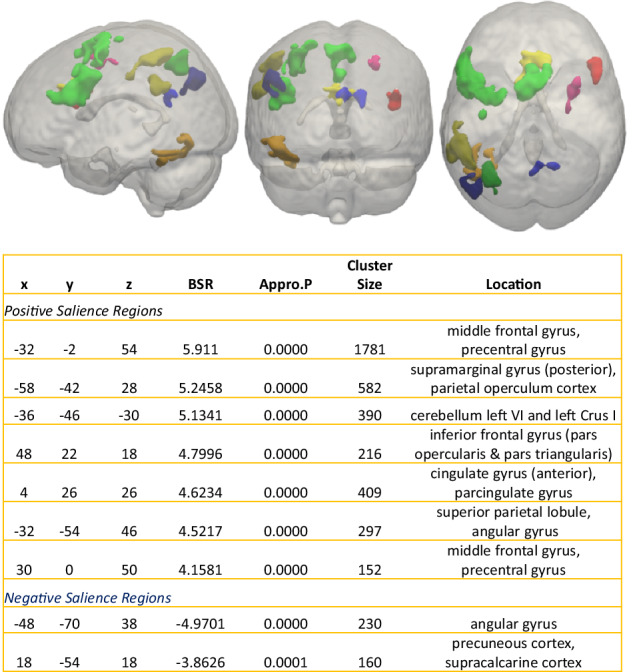
Condition-by-timepoint effects, lag 6. Clusters where peak voxels have a salience/standard error ratio (i.e. Bootstrap ratio; BSR) of ≥3 or ≤−3 (p < 0.001) for the latent variable (LV) describing 100% of all the variance for our desired contrast comparing conditions and timepoints. Yellow, green, red and pink clusters (i.e., positive salience regions) indicate increased activation during tDCS compared to sham for the incongruent compared to the congruent condition. Blue clusters (i.e., negative salience regions) illustrate increased activation for the opposite contrast (i.e., increased activation during sham compared to tDCS for the incongruent compared to the congruent condition). Lag 6 represents the brain activity at 6 TRs post stimulus onset (this sequence had a TR of 1 s). n = 47 subjects. Results based on lags 5, 7, and 8 are reported in the supplementary material (figure S2).

To answer our main research question (i.e., how tDCS stimulation affects brain activation patterns during inhibition performance), in a second step, the interaction between condition (i.e., incongruent > congruent) and timepoint (i.e., tDCS > sham) was investigated. The PLS found a significant interaction-LV (p < 0.034) with positive brain scores for stimulation-incongruent and sham-congruent conditions, indicating increased activation during tDCS compared to sham for the incongruent compared to the congruent condition (Fig. 4). The opposite contrast (i.e., increased activation during sham compared to tDCS) did not display any significant results.

### Mediation analysis

**Fig. 5:**
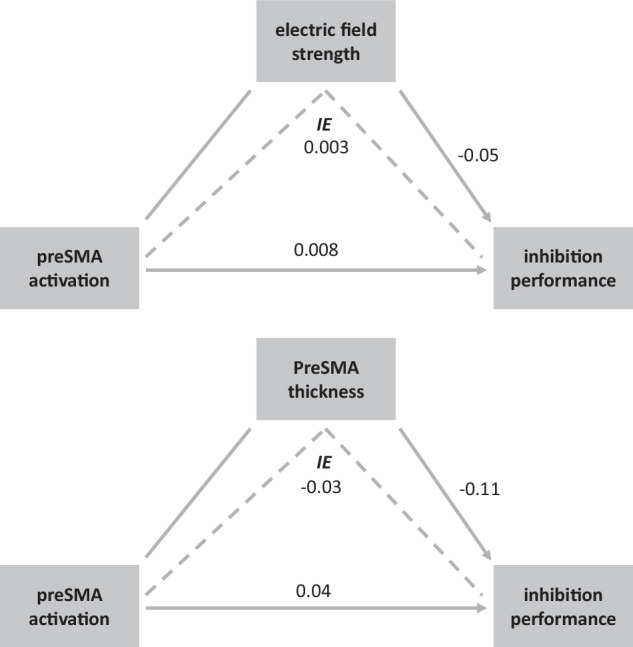
Mediation Analysis investigating potential effects of electric field strength and right PreSMA thickness (n = 47). The p-values are depicted on the lines linking the associations and mediations.

Results of the mediation analysis with EF strength as the mediating variable showed no association between stimulation-related changes in brain activation and stimulation-related changes in inhibition performance (β = 0.008, n.s.), no association between stimulation-related changes in inhibition performance and EF strength (β = −0.05, n.s.), and no indirect association between stimulation-related changes in brain activation and stimulation-related changes in inhibition performance (i.e., mediated by EF strength) (β = 0.003, n.s.) (Fig. 5). The GFI of the mediator ROI model was 0.99, indicating an adequate model fit.

Results of the mediation analysis with right pre-SMA thickness as the mediating variable showed no association between stimulation-related changes in brain activation and stimulation-related changes in inhibition performance (β = 0.04, n.s.), no association between stimulation-related changes in inhibition performance and thickness (β = −0.11, n.s.), and no indirect association between stimulation-related changes in brain activation and stimulation-related changes in inhibition performance (i.e., mediated by thickness) (β = −0.03, n.s.) (Fig. 5). The GFI of the mediator ROI model was 0.96, likewise indicating an adequate model fit.

Finally, the assessment of the mean EF intensity for 31 ROIs as described in the DKA and 9 ROIs as described in the HMAT atlas showed strongest intensities around our target region, the pre-SMA, as well as moderate to weak intensities in more distant areas (Fig. 5).

## Discussion

### Behavioural and brain activity results

In the current study we investigated whether tDCS over the pre-SMA was able to improve inhibition performance in OCD patients, as had been previously observed in healthy participants [21]. Irrespective of stimulation timepoint, patients performing the Stroop task displayed a significant increase in percentage of correct responses during stimulation in comparison to sham. On a cerebral level, this improved inhibition performance during tDCS compared to sham was associated with an increased activation in a fronto-parieto-cerebellar network comprising, amongst others, the pre-SMA, the IFG, the anterior cingulate, the superior parietal lobe and parts of the cerebellum. Hence, present findings indicate tDCS’s ability to improve inhibition performance in OCD which has been shown to be impaired in patients [18, 50, 51]. These inhibitory improvements are expected to be sustained after multiple sessions of stimulation, as also demonstrated by a recent study targeting the preSMA [52]. It should be noted, however, that performance improvements were only noticeable with regard to the percentage of correct responses. We consider it plausible that improvement in response time unlike inhibition accuracy could require multiple tDCS sessions since slower responding reflecting hesitant response behaviour might be more closely linked to the clinical state of the patients. In general, OCD patients tend to display a larger speed variability as well speed underperformance in inhibition tasks [53]. Additionally, studies have reported that OCD patients tend to exhibit post-error slowing to improve their performance accuracy [50, 54]. We assume that this prioritization of accuracy over speed, which is also closely related to an excessive error-monitoring [50, 55] and reflects inhibited, hesitant response behavior, might only change when tDCS effects exert a significant influence on the clinical state of the patients (i.e., which is usually the case after 10-20 stimulation sessions).

There are surprisingly few fMRI-tDCS concurrent studies in OCD patients, despite the field moving towards this format [56–58]. The present study which is, to our best knowledge, the first study applying concurrent tDCS-fMRI in patients with OCD indicates that a 2 mA single-session tDCS (compared to sham) over the pre-SMA induces activation increases in, amongst others, the pre-SMA and the IFG and thus in areas that have previously been shown to be altered during inhibition tasks in OCD patients [20]. In addition, present findings show a tDCS-associated activation increase in several regions that a large meta-analysis [15] found to be decreased in OCD patients during inhibition. These regions contain the cingulum, the orbital frontal cortex, the IFG, the cerebellum, and the angular gyrus. Consequently, our findings provide first and, preliminary, evidence that tDCS stimulation of the pre-SMA and neighbouring regions (Fig. 2) can improve inhibition performance and normalize altered neuronal activity in patients with OCD. Two additional aspects should not go unmentioned. First, the opposite contrast outlining the contrast sham>stimulation for incongruent>congruent was not significant. And, second, our PLS analysis contrasting the incongruent with the congruent condition independent of stimulation condition showed significant activation in several regions (i.e., the cingulum, the inferior/middle/superior frontal gyrus, and the superior parietal gyrus) that have been demonstrated by a recent meta-analysis [14] to be involved in inhibition performance in healthy young individuals and which partly showed an additional increase in activation during tDCS. Together, our findings in association with the results of previous studies indicate that 1., in general, patients with OCD seem to employ regions and networks during inhibition that are partly overlapping with those regions found to be activated in healthy young individuals during these processes, 2. activity in some – mainly motor-related - regions [59–61], some of which have previously been shown to exhibit a decreased activity in OCD during inhibition, seem to experience an increase in activation during tDCS, and 3. this activation increase might constitute the mechanism enabling a significantly improved behavioural performance.

### Pre-SMA thickness and electric field

Given previous studies showing that EF strength and individual brain anatomy (cortical and skull thickness) are critical determinants of the final stimulation effects [24–28] we investigated a potential influence of these parameters by means of a mediation analysis. We found no mediation with EF strength as the mediating variable between pre-SMA activation and inhibition performance. The same was observed when examining a potential mediating effect of pre-SMA cortical thickness. Additionally, the mediation analysis found no direct association between any of the individual factors. One reason for the lack of association might be that tDCS does not exert very focal stimulation effects. As illustrated in Fig. 2, although the strongest EF intensities are expectedly detectable around the stimulation target region, i.e., the pre-SMA, various additional areas present at least moderate or light EF intensities. Hence, pre-SMA activation or thickness might not be indicative of overall association between stimulation-driven underlying brain activity and inhibition performance.

Finally, the question of whether stimulation efficacy is driven by brain-state [62, 63] or by the angle and intensity [28, 64, 65] at which the current traverses neurons in the grey matter continues to be unresolved. Modelling of the EF field has recently gained a lot of popularity to better account for interindividual changes in brain anatomy. This study shows that modelling alone does not necessarily answer this question. Studies with larger sample sizes and a decreased interindividual variability in brain anatomy might be necessary to further elucidate this.

### Inhibition as a measure of stimulation efficacy

tDCS effects are said to last between 20 to 30 min after a single session, dependent on session duration [66, 67]. For sustained OCD symptom alleviation at least 20 repeating sessions are alleged to be necessary [68–70]. Therefore, we expected to not find any significant improvement in OCD symptoms. Rather, this study aimed to investigate the underlying neuronal correlates responsible for the plasticity changes during long-term tDCS intervention, by examining short term changes in brain activation or, broadly speaking, the general mechanisms underlying the effects of the stimulation. Given our set-up (i.e., concurrent tDCS-fMRI) we believe that current findings contribute to a better understanding of these mechanisms, predominantly since depolarisation changes in a target region of tDCS can lead to increased activation in an extended network best identified during simultaneous stimulation.

### Limitations

The choice of utilising an inhibitory task as an indicator of intervention efficacy was due to inhibition deficits being assumed to constitute a core characteristic, largely independent of clinical profile, within OCD [51]. For this reason, the study did not differentiate between symptom profiles when including subjects in the study. Nevertheless, symptom heterogeneity in our OCD sample should be mentioned as a limitation since in cannot be excluded that tDCS effects differ depending on the individual symptom profile. Equally, the study recognises that in including both medicated and non-medicated patients it cannot preclude medication effects on tDCS response. Furthermore, a multi-session tDCS study following our parameters would allow us to explore the connectivity and behavioural effects over time, which would be beneficial for investigating future therapeutic application. Another essential exploration would be including a healthy control group in the study to juxtapose the patient findings detailed in this study.

## Conclusion

Anodal tDCS in OCD patients can successfully improve inhibition performance in the Stroop task when targeting the pre-SMA for a 20-min stimulation duration. In the field’s continued effort to establish different types of transcranial electric stimulation (tES) as non-invasive therapeutic interventions, future studies should aim to further investigate the relevance of cortical thickness, EF patterns, as well as brain activity and state during stimulation in order to increase our knowledge about the mechanisms underlying tES to prospectively establish optimized tES treatment schemes for different psychiatric disorders.

## Supplementary information

Figure S1

Figure S2

Figure S3a

Figure S3b

Supplementary Material Figure and table legends

table S1

## Data Availability

The datasets generated during and/or analysed during the current study are available from the corresponding author on request.
