# Supplementary figures and images for: Investigating the effects of brain stimulation on the neural substrates of inhibition in patients with OCD: A simultaneous tDCS – fMRI study
Source: Transl Psychiatry. 2025 May 19;15:173. doi: 10.1038/s41398-025-03381-9 (PMC12089465; doi:10.1038/s41398-025-03381-9)

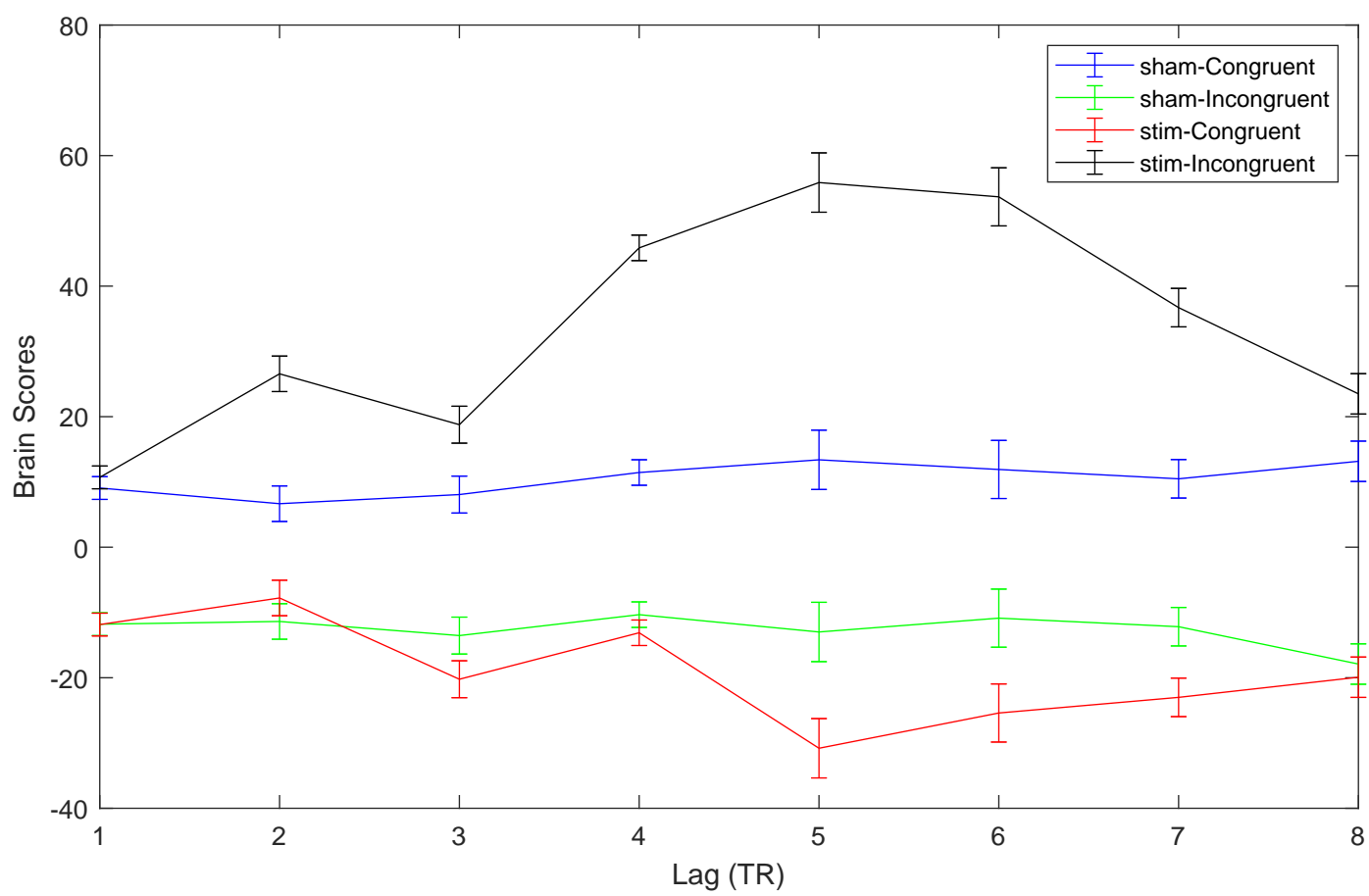

Supplement: Supplementary file 1 — Figure S1 [file 41398_2025_3381_MOESM1_ESM.pdf]

Density

0.2

0.3

0.4

0.5

0.6

0.7

0.8

V/m electric field

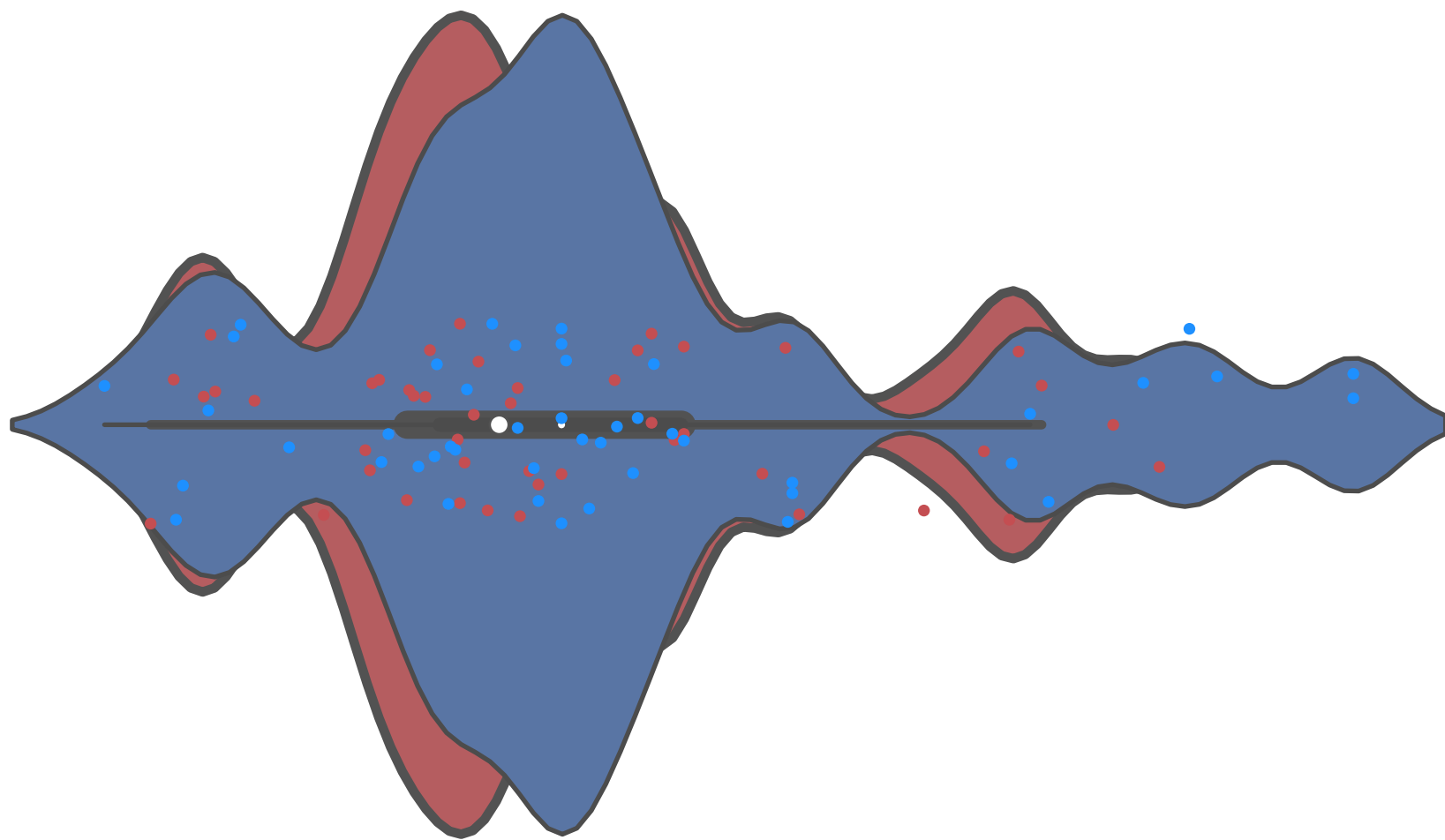

Supplement: Supplementary file 3 — Figure S3a [file 41398_2025_3381_MOESM3_ESM.pdf]

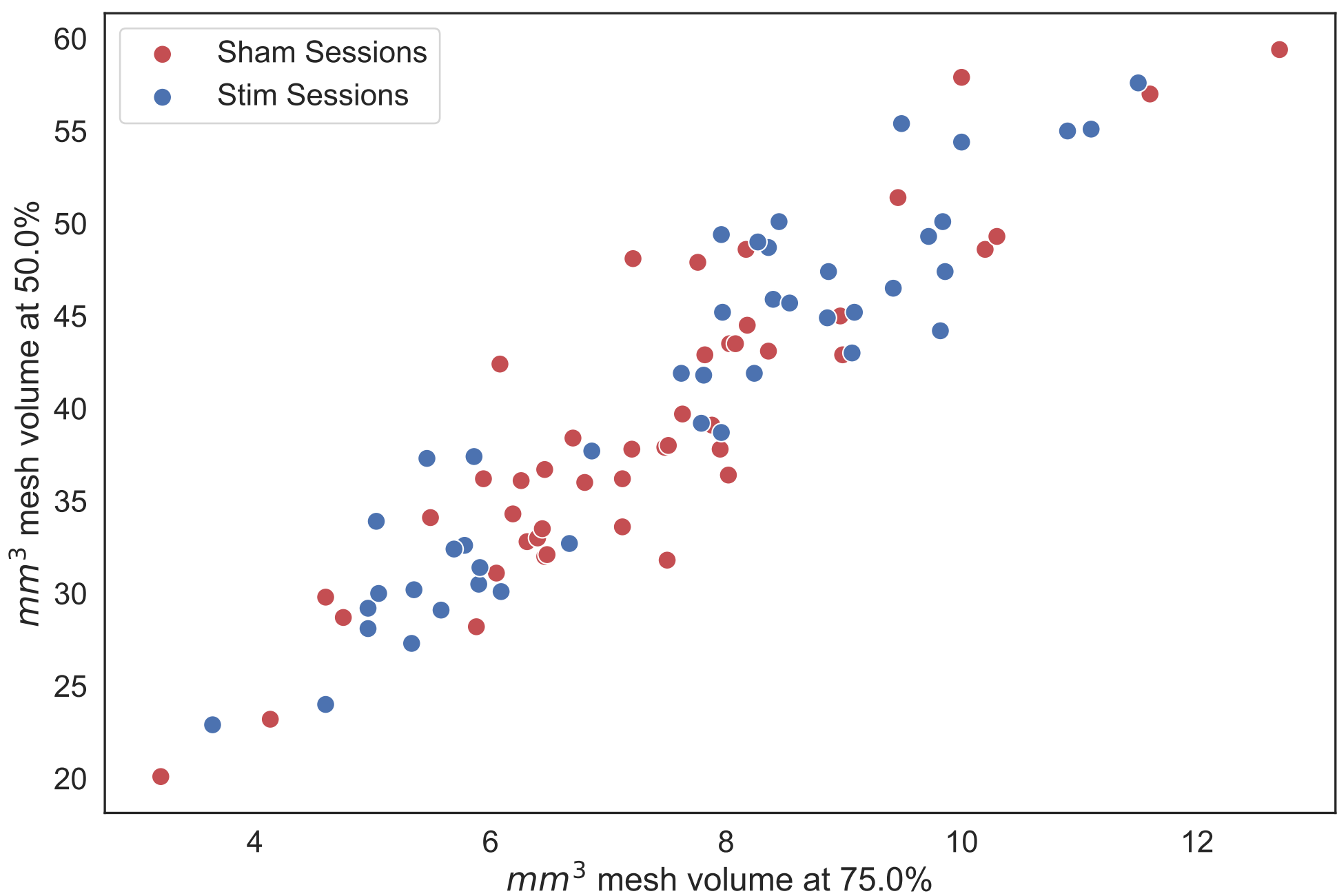

Supplement: Supplementary file 4 — Figure S3b [file 41398_2025_3381_MOESM4_ESM.pdf]
