# Supplementary material for: Investigating the effects of brain stimulation on the neural substrates of inhibition in patients with OCD: A simultaneous tDCS – fMRI study
Source: Transl Psychiatry. 2025 May 19;15:173. doi: 10.1038/s41398-025-03381-9 (PMC12089465; doi:10.1038/s41398-025-03381-9)
Supplement: Supplementary file 2 — Figure S2 [file 41398_2025_3381_MOESM2_ESM.ppt]

## Slide 1
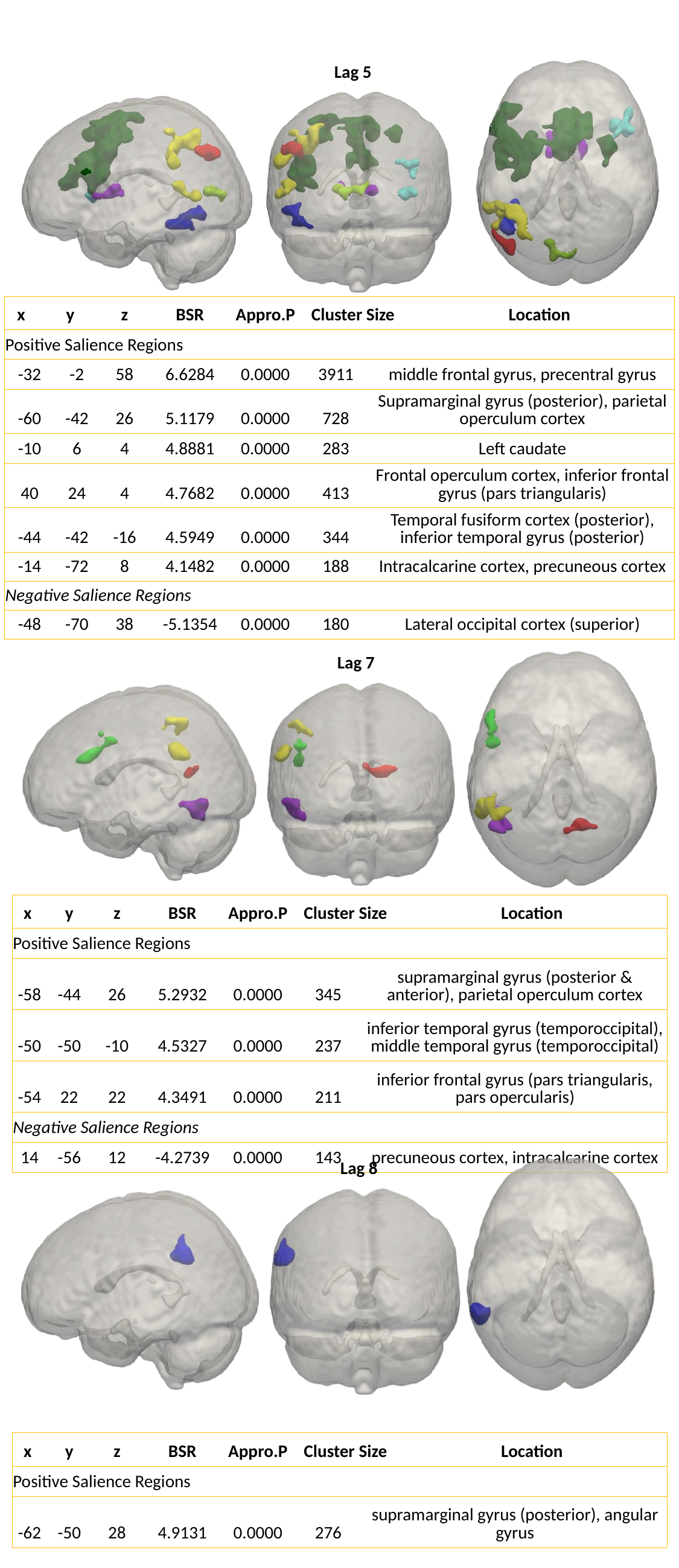

Lag 5
| x | y | | z | BSR | Appro.P | Cluster Size | | Location |
| --- | --- | --- | --- | --- | --- | --- | --- | --- |
| Positive Salience Regions | | | | | | | | |
| -32 | | -2 | 58 | 6.6284 | 0.0000 | 3911 | middle frontal gyrus, precentral gyrus | |
| -60 | | -42 | 26 | 5.1179 | 0.0000 | 728 | Supramarginal gyrus (posterior), parietal operculum cortex | |
| -10 | | 6 | 4 | 4.8881 | 0.0000 | 283 | Left caudate | |
| 40 | | 24 | 4 | 4.7682 | 0.0000 | 413 | Frontal operculum cortex, inferior frontal gyrus (pars triangularis) | |
| -44 | | -42 | -16 | 4.5949 | 0.0000 | 344 | Temporal fusiform cortex (posterior), inferior temporal gyrus (posterior) | |
| -14 | | -72 | 8 | 4.1482 | 0.0000 | 188 | Intracalcarine cortex, precuneous cortex | |
| Negative Salience Regions | | | | | | | | |
| -48 | | -70 | 38 | -5.1354 | 0.0000 | 180 | Lateral occipital cortex (superior) | |
Lag 7
| x | y | z | BSR | Appro.P | Cluster Size | | Location |
| --- | --- | --- | --- | --- | --- | --- | --- |
| Positive Salience Regions | | | | | | | |
| -58 | -44 | 26 | 5.2932 | 0.0000 | 345 | supramarginal gyrus (posterior & anterior), parietal operculum cortex | |
| -50 | -50 | -10 | 4.5327 | 0.0000 | 237 | inferior temporal gyrus (temporoccipital), middle temporal gyrus (temporoccipital) | |
| -54 | 22 | 22 | 4.3491 | 0.0000 | 211 | inferior frontal gyrus (pars triangularis, pars opercularis) | |
| Negative Salience Regions | | | | | | | |
| 14 | -56 | 12 | -4.2739 | 0.0000 | 143 | precuneous cortex, intracalcarine cortex | |
Lag 8
| x | y | z | BSR | Appro.P | Cluster Size | | Location |
| --- | --- | --- | --- | --- | --- | --- | --- |
| Positive Salience Regions | | | | | | | |
| -62 | -50 | 28 | 4.9131 | 0.0000 | 276 | supramarginal gyrus (posterior), angular gyrus | |
