# Supplementary material for: Investigating the effects of brain stimulation on the neural substrates of inhibition in patients with OCD: A simultaneous tDCS – fMRI study
Source: Transl Psychiatry. 2025 May 19;15:173. doi: 10.1038/s41398-025-03381-9 (PMC12089465; doi:10.1038/s41398-025-03381-9)
Supplement: Supplementary file 5 — Supplementary Material Figure and table legends [file 41398_2025_3381_MOESM5_ESM.docx]

Supplementary Material Legends

Table S1: TDCS stimulation side effects

Figure S1a: Electric Field magnitude at 99.9% percentile of vector fields

Figure S1b: Mesh volume (mm^3) at 50% and 75% of the 99.9^th^ percentile electrical field

Figure S2: Brain Scores of condition-by-timepoint effects at different acquisition timepoints after the stimulus. (TR = 1 second or our sequence)

Figure S3: Condition-by-timepoint effects at lags 5, 7 and 8
